# Nodding behavior couples to vigilance fluctuation in a high-calorie diet model of drowsiness

**DOI:** 10.1186/s13041-018-0377-4

**Published:** 2018-06-07

**Authors:** Anna Shin, Jeonghoon Woo, Jung Eun Kim, Daesoo Kim

**Affiliations:** 1 Department of Biological Sciences, Korea Advanced Institute of Science and Technology (KAIST), Daejeon, 34141 Republic of Korea; 2 Chungnam Techno Park, Cheonan, 31035 Republic of Korea

**Keywords:** Drowsiness, Vigilance dynamics, Head nodding, High-fat food, Delta oscillation, Cav3.1-knockout mice

## Abstract

Drowsiness is an awake state with increased sleep drive, yet the neural correlates and underlying mechanisms remains unclear. Here, we established a mouse model of drowsiness, where mice are fasted for 1 day and then allowed to overeat high-fat food (to promote sleep) while positioned in an open-field box (to promote vigilance). They fall into a long-lasting drowsy state, as reflected by repeated and open-eyed nodding of the head while in a standing position. Simultaneous recording of electroencephalogram (EEG) and neck electromyogram (EMG) readouts revealed that this drowsy state including nodding state had multiple stages in terms of the relationship between the level of vigilance and head movement: delta oscillations decreased in power prior to the head-nodding period and increased during the non-nodding period. Cav3.1-knockout mice, which have reduced delta oscillations, showed frequent head nodding with reduced duration of nodding episodes compared to wild-type mice. This suggests that the balance of drive is tilted in favor of wakefulness, likely due to their previously proposed decrease in sleep-promoting functions. Our findings indicate that delta oscillations play a dominant role in controlling vigilance dynamics during sleep/wake competition and that our novel mouse model may be useful for studying drowsiness and related neurological disorders.

**Electronic supplementary material:**

The online version of this article (10.1186/s13041-018-0377-4) contains supplementary material, which is available to authorized users.

## Introduction

In the drowsy state, sleep and wake drives coexist to allow animals to maintain a minimal vigilance that can allow them to avoid potential risks, such as predatory attacks [1, 2]. However, it has proven challenging to study the mechanisms underlying sleep/wake competition in the drowsy state, largely because: 1) drowsiness occurs intermittently with short latency, meaning that there is only a limited time for mechanistic observation; and 2) due to stress, animals remain vigilant in experimental test boxes even during their normal sleep cycle. Thus, we need to establish experimental conditions that will reliably induce a sustained drowsiness during waking state.

T-type Ca^2+^ channels (Cav3) have been associated with both sleep and wake drive. According to the sleep oscillation hypothesis, T-type Ca^2+^ channels play a relevant role in sleep consolidation by generating burst firing during sleep in thalamic neurons to yield sleep oscillations such as sleep spindles and delta oscillations in the thalamocortical pathway during NREM sleep [3–5]. Indeed, mice lacking the Cav3.1 gene (Cav3.1-KO), which encodes for the alpha subunit of T-type Ca^2+^ channels, lack thalamic burst firing and show unstable NREM sleep [6, 7]. In contrast, the wake-up call hypothesis [8] proposes that thalamic burst firing attributed to T-type Ca^2+^ channels strongly stimulates the cortex to increase attention or vigilance. Indeed, lateral geniculate nucleus (LGN) neurons show burst firing in response to light stimuli during the partially anesthetized or waking state in cat [9, 10] probably through a strong post-synaptic impact on cortical neurons [11].

However, identifying the role of T-type Ca^2+^ channels during the waking state has been challenging because thalamic burst firing is rarely observed during wakefulness [12]. To address this issue, we first tried to establish a rodent model of drowsiness, a waking state, in which delta oscillations emerge [13, 14] and compared behavioral and electroencephalogram (EEG)/electromyogram (EMG) activity between WT and Cav3.1-KO mice.

## Results

### Facilitating induction of nodding by high-calorie feeding in an open-field box

**Fig. 1:**
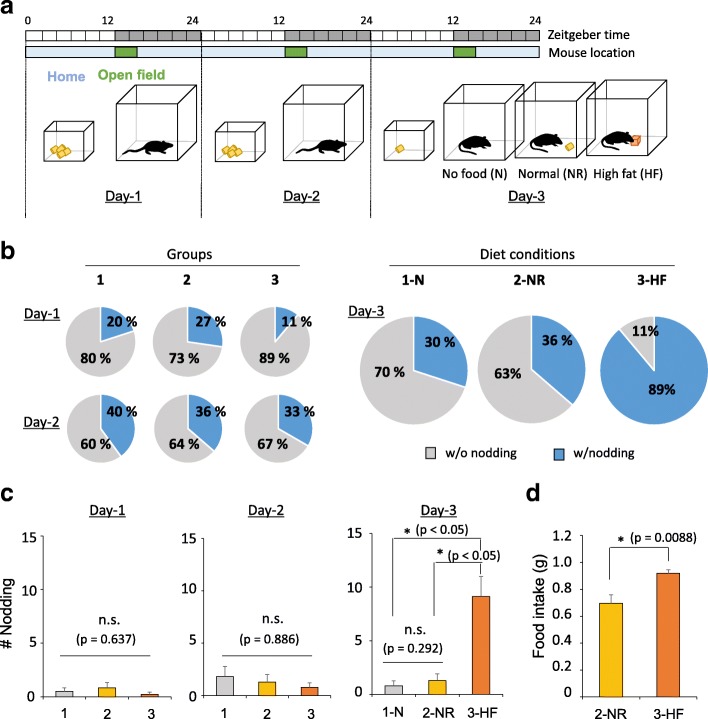
Overeating in an open-field box increases head-nodding behavior. **a** A schematic depiction of the new drowsiness model induced by food in an open-field box. One group was not provided with food while the other groups consumed SPAM as a high-fat food or normal food. **b** The ratio of head nodding behavior showing mice in the no-food, normal food and high-fat food groups during each day (no food; *n* = 10, normal food; *n* = 11, high-fat; *n* = 9). **c** The number of head nodding was not different among the groups during day-1 and day-2 (one-way ANOVA on ranks, *p* = 0.637 and *p* = 0.886, no food; *n* = 10, normal food; *n* = 11, high-fat; *n* = 9). Mice in the high-fat food group showed frequent head nodding during day-3 (one-way ANOVA on ranks, *p < 0.05 and *p* = 0.292, Dunn’s post hoc test, n.s. indicates ‘not significant’). All error bars represent s.e.m. **d** Quantification of consumed food between normal food and high-fat food groups (Mann-Whitney U test, *p* = 0.0088, normal food; *n* = 11, high-fat; *n* = 9). All error bars represent s.e.m.

We designed an experimental procedure to induce sleep and wake drives during the same time in C57BL/6 J mice (Fig. 1) based on the rationale that a high caloric state may increase sleep drive [15, 16] and exposure to an open-field may increase vigilance [17] . During day-1 and -2, mice were fed normal food ad libitum in their home cage and put into an open-field box for 1.5 h to measure their basal state. At the end of the day-2 open-field session, the mice were given only 0.5 g food to induce overeating the next session. On day-3, mice were divided into three groups and given either no food (N), 1.5 g of normal food (NR) or high fat food (HF) (Fig. 1a). These three group of mice were individually exposed to open-field box for 1.5 h.

First, we defined behavioral characteristics according to vigilance states, such as wake, sleep and drowsiness (Additional file 1: Figure S1). For example, mice showed no body movements (“freezing”) and closed eyes during sleep, whereas they showed dynamic head and body movements for wakefulness. In addition, some mice showed a drowsy state which included head nodding (Nd) behavior with opened eyes in a standing posture (not lying on the ground and showing no dynamic locomotion). Between Nd behaviors, they showed non-nodding state (Non-Nd), in which there is no head movement with opened eyes (Additional file 1: Figure S1). The number of mice showing head nodding increased on day-2 compared to the day-1 open-field session (Fig. 1b) although the number of head nods per mouse was not significantly different between groups during the day-1 and -2 open-field session (Fig. 1c). During the day-3 open-field session after different diet conditions (N, NR, HF), around 89% of the mice in the HF group showed head nodding whereas only 30–36% of those in the N or NR groups did (Fig. 1b). These HF mice showed a higher number of nodding during the open-field session than other groups (Fig. 1c). Considering that they had increased food intake (Fig. 1d), the high-calorie state significantly increases sleep drive during the waking state and increases head nodding in an open-field box. However, the mean duration of each nodding episode and detailed behavioral stages therein was not significantly different between N and NF mice (Additional file 2: Figure S2). Thus, HF diet increases the total period of head nodding by increasing the incidence thereof.

### Characteristics of EEG and EMG during nodding behavior

**Fig. 2:**
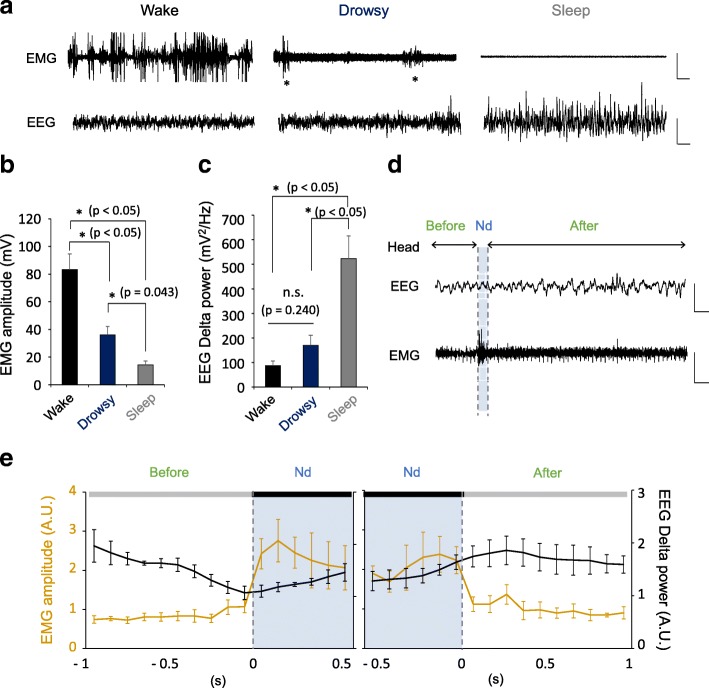
The properties of delta oscillation and EMG amplitude during drowsy state including nodding behavior. **a** Representative traces of EMG/EEG signals obtained during wake, drowsy and sleep state. Scale bars, 400 mV, 2 s for EMG and 100 mV, 2 s for EEG. **b** The average of EMG amplitude in wake, drowsy and sleep state (repeated one-way ANOVA, *p < 0.05 and *p* = 0.043, Sidak’s post hoc test, *n* = 8). All error bars represent s.e.m. **c** Quantification of EEG delta power during wake, drowsy and sleep state. (repeated one-way ANOVA, *p < 0.05 and *p* = 0.240, Sidak’s post hoc test, *n* = 8, n.s. indicates ‘not significant’). All error bars represent s.e.m. **d** Representative EEG and EMG traces obtained during drowsy states. The drowsy state consisted of sequential Nd and Non-Nd (‘before’ and ‘after’ period of Nd state) of the head (Nd; nodding, Non-Nd; non-nodding). Scale bars, 100 mV, 1 s for EEG and 50 mV, 1 s for EMG. **e** *Left:* In the course of changing from the Non-Nd state to the Nd state, the EMG signal increased and delta oscillation decreased. *Right:* The graph during Nd state to Non-Nd state showing increase of EEG delta power and decrease in EMG amplitude (*n* = 4)

We investigated the vigilance states of HF mice in open field (Fig. 2). During the drowsy state (Nd + Non-Nd), they showed neck EMG activity only during the Nd stage but this activity disappeared during the Non-Nd stage (Fig. 2a). The EMG pattern during the drowsy state was easily discernable from the waking or sleep state, which is coupled with vigorous or silent EMG activities respectively (Fig. 2b). EEG analysis of delta oscillations, a measure of sleep drive, revealed that the averaged power of delta oscillations during the drowsy state is lower than that during the sleep state, but was not statistically different with that during the waking state (Fig. 2a, c). For more detailed analysis, we compared the EEG fluctuations with the transition between Non-Nd and Nd states (Fig. 2d) and found that the power of delta oscillations smoothly decreased prior to the Non-Nd (Before-Nd)➔Nd transition and increased during the Nd➔ Non-Nd (After-Nd) transition (Fig. 2e). These results suggest that head nodding is coupled with vigilance fluctuations, probably due to the competition between sleep and wake drives.

### Analysis of nodding behavior in Cav3.1-KO mice

**Fig. 3:**
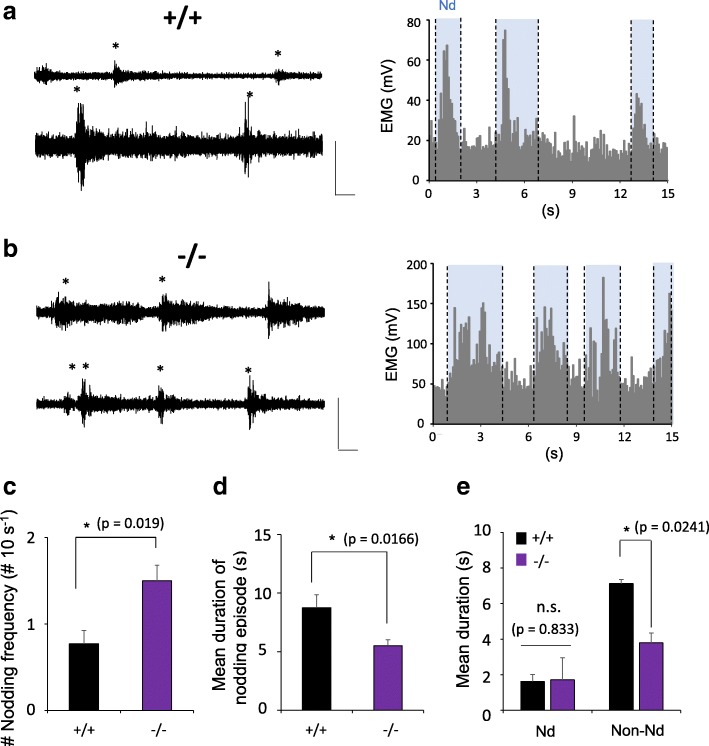
Knockout of Cav3.1 in the thalamus is associated with a higher vigilance level during drowsy state including nodding behavior. **a** *Left:* Typical EMG sample traces from two mice in Cav3.1^+/+^ group during drowsy state including nodding behavior. *Right:* Graph showing the EMG duration and amplitude of Nd state in Cav3.1^+/+^ mice. Asterisks represents Nd states. Scale bars, 200 mV, 1 s. **b** Representative EMG traces from two mice in Cav3.1^−/−^ group during drowsy state including nodding behavior. The Cav3.1^−/−^ group showed a higher frequency of head nodding behavior. Scale bars, 200 mV, 1 s. **c** The frequency of head nodding averaged over 10 s is higher in Cav3.1^−/−^ mice (Mann-Whitney rank sum test, *p* = 0.019; *n* = 4 for Cav3.1 ^+/+^ and *n* = 6 for Cav3.1^−/−^). All error bars represent s.e.m. **d** The duration of nodding episode (mean duration of single Nd + Non-Nd state) was different between Cav3.1^+/+^ and Cav3.1^−/−^ groups (unpaired t-test, *p* = 0.0166; *n* = 4 for Cav3.1^+/+^ and *n* = 6 for Cav3.1^−/−^). All error bars represent s.e.m. **e** Quantification of mean duration between Cav3.1^+/+^ and Cav3.1^−/−^ during Nd and Non-Nd states (unpaired t-test, *p* = 0.833 and *p* = 0.0241; *n* = 4 for Cav3.1^+/+^ and *n* = 6 for Cav3.1^−/−^, n.s. indicates ‘not significant’). All error bars represent s.e.m.

To prove the sleep-wake competition hypothesis, we examined the drowsy state, including head nodding behavior, in Cav3.1-KO mice, which are known to show higher vigilance, characterized by resistance to pharmacologically induced absence seizures [6] and more frequent awakenings under anesthesia [18]. During the HF-induced drowsy state, Cav3.1-KO mice showed more frequent nodding behavior than WT mice (Fig. 3a, b, c) and shorter duration of nodding episode (duration of single Nd + Non-Nd) than WT (Fig. 3d). There was no differences in the duration of Nd state between genotypes (Fig. 3e) but the duration of Non-Nd state decreased in Cav3.1-KO mice (Fig. 3e). This finding shows that Cav3.1-KO mice show wake-up like behavior with frequent Nd state and reduced sleep-like state with short non-Nd state, supporting the idea that T-type Ca^2+^ channels promote sleep during the drowsy state, which has been a controversial issue. Altogether, our results suggest that head nodding behavior robustly correlates with vigilance fluctuations resulting from competition between sleep and wake drives.

## Discussion

Here, we established a behavioral paradigm for inducing a long-lasting drowsiness characterized by frequent nodding behaviors (Fig. 1). To increase the sleep drive, we fasted mice and then provided them with HF for overeating, and successfully induced head nodding in an open-field box (Fig. 1a). The head nodding state is different from sleep and freezing. In the case of sleep, the eyes are closed and the body takes a crouching posture. “Freeze” is defined as becoming almost totally motionless, but remaining standing, sometimes on two legs. The eyelids are strongly retracted, in contrast with their half-closed position during relaxed wakefulness. In addition to the behavioral difference, drowsy EEG and the EEG patterns of sleep/freezing are quite different. Sleep is characterized by high voltage and low frequency whereas freezing has the characteristic of low voltage fast activity [19]. Our strategy for making a nodding behavioral model takes advantage of the previous findings that: the metabolic state is deeply related to vigilance states [20, 21]; a rise in glucose concentration due to overeating promotes sleep by increasing the activity of VLPO neurons [22]; and that orexinergic neurons involved in arousal maintenance are inhibited by an increase in the extracellular glucose concentration [23, 24]. To increase vigilance in the HF mice, we placed them in an open field, which is known to induce mild stress and anxiety [17]. As people who suffer from persistent stress or excessive anxiety typically have sleeping issues, such as insomnia [25], we expected stress and anxiety to interfere with the ability of our model mice to fall asleep.

Next, we analyzed the physiological characteristics of nodding behavior based on EEG and EMG. We characterized the dynamics of delta power of EEG and EMG amplitudes during the drowsy state including Nd and Non-Nd state. In the course of changing from Non-Nd state to Nd state, EMG signal increased and delta oscillation decreased. And in the case of Nd state to Non-Nd state, EEG delta power increased and EMG amplitude decreased (Fig. 2e). Thus, this drowsy state is a dynamic state that is includes transitions of sleep-like Non-Nd state and wake-like Nd state. Furthermore, we analyzed the effects of HF on the properties of each nodding state. We found that there was no statistically significant difference in the duration of episodic nodding, Nd and Non-Nd state between N and HF mice groups (Additional file 2: Figure S2a, b). According to these results, we confirmed that overeating in an open field increases the frequency of nodding behaviors but does not change the characteristics of drowsy state including nodding behavior.

Cav3.1-KO mice, which are known to have reduced delta oscillations, have a higher tendency for vigilance in the nodding state compared to WT mice (Fig. 3). In our experiments, Cav3.1-KO mice exhibited more frequent Nd with shorter Non-Nd duration than WT mice (Fig. 3c, e), supporting the previous suggestion that T-type Ca^2+^channels promote sleep drive [6, 26]. Many neurological disorders are associated with an imbalance between sleep and vigilance. Hypervigilance is a state of increased alertness and tension that has been associated with PTSD, anxiety, schizophrenia [27] and insomnia [25]. If waking dominates the competition in the drowsy state, people will have trouble falling or staying asleep (insomnia). This insomnia afflicts patients not only at nighttime but also gives rise to daytime symptoms. In contrast, patients with hypersomnia consistently feel drowsy during the day, which causes excessive daytime sleepiness [28], and oversleeping (a state with reduced wake-up efficiency, symptoms frequently reported in patients with depression [29, 30]). As shown here using Cav3.1-KO mice, our new mouse model of drowsiness provides a good opportunity to demonstrate the interaction between sleep and arousal mechanisms during waking state and in related neurological disorders.

## Methods

### Animals

All behavioral experiments were performed on 9- to 11-week-old male C57BL/6 J, 11- to 13-week-old male Cav3.1^+/+^ and Cav3.1^−/−^ C57BL/6 J mice. Mice heterozygous for the Cav3.1 gene (Cav3.1^+/−^) were generated using embryonic stem cells of the 129/Sv genetic background [7, 26]. To make the C57BL/6 J congenic strain, F1 heterozygous mice obtained from mating chimera and C57BL/6 J females were backcrossed with C57BL/6 J mice for ≈25 generations (N25). Cav3.1^−/−^ mice and WT littermates obtained from mating between C57BL/6 J (N25) Cav3.1^+/−^ mice were used for this study. Mice had free access to food and water and were kept on a 12-h light-dark cycle at 22 °C. The care and handling of mice was performed according to the directives of the Animal Care and Use Committee of KAIST (Daejeon, Korea).

### Overeating protocol for head-nodding behavior

We performed all of the experiments during the dark period (ZT12~ZT13.5 or ZT13.5~ZT15) for 3 days. On the first and second day, each mouse was placed in a soundproof chamber (inner size: 25 cm * 30 cm * 40 cm) and video recording for behavioral analysis was conducted for 1.5 h (ZT12~ZT13.5 or ZT13.5~ZT15). After the second day of recording, each mouse was returned to a home cage with only 0.5 g of food. On the third and final day, no food was served for mice group 1, normal chow (1.5 g) for group 2, SPAM (Hormel Foods Corporation, USA) paste (1.5 g) for group 3. For EEG/EMG recording, we first performed surgery for EEG/EMG electrode implantation. After 5~ 7 days of surgical recovery, EEG/EMG signal detection and video recording were performed simultaneously for 3 days during the dark period (ZT12~ZT13.5 or ZT13.5~ZT15), as with the above protocol.

### Behavioral analysis of drowsiness

The drowsy state was defined by video and EMG recording data. The head-nodding and non-nodding behavior was categorized with video analysis and the oscillation of muscle tone on EMG.

### EMG and EEG

EEG and EMG signals were amplified with a Grass model 9E polygraph (Grass Technologies) and digitized at a sampling rate of 2 kHz (DIGIDATA 1320A; Molecular Devices). Four epidural electrodes were implanted [1.4 mm anteroposterior (AP), ±1.3 mm mediolateral (ML), and 2.4 mm AP, ML ±2.4 mm ML from bregma], and a reference electrode was implanted in the cerebellar region of the skull. EMG amplitude and video data were analyzed to discriminate each behavioral states. To compare the EEG and EMG fluctuations with the transition between Non-Nd and Nd states, delta power was analyzed through short time fourier transform, and EMG was smoothed to match the power analysis. Delta power and EMG was normalized by the mean value of nodding episode.

### Data analysis

EEG signals were high-pass-filtered at 0.7 Hz to eliminate movement artifacts. Short-Time Fourier Transform (STFT) was applied to examine the EEG power density as a function of time. The signals were analyzed using custom MATLAB code (MathWorks, USA).

## Additional files

Additional file 1: **Figure S1.** Behavioral measurement according to vigilance level. Comparison of head/eye condition according to the behavior patterns. (PDF 92 kb)

Additional file 2: **Figure S2.** Analysis of HF effects on drowsy states including nodding behavior. (a) The average length of nodding episode in the no-food and high-fat food groups (unpaired t-test, *p* = 0.887, no food *n* = 2 and high-fat *n* = 4, n.s. indicates ‘not significant’). All error bars represent s.e.m. (b) The mean duration of no food and high-fat food groups during Nd (unpaired t-test, *p* = 0.886) and Non-Nd states (unpaired t-test *p* = 0.895, no food *n* = 2 and high-fat *n* = 4). All error bars represent s.e.m. (PDF 88 kb)

## Abbreviations

Cav3.1^−/−^: Mice null for Cacna1g
EEG: Electroencephalogram
EMG: Electromyography
LGN: Lateral geniculate nucleus
NREM: Non-rapid eye movement
nRT: Thalamic reticular
VLPO: Ventrolateral preoptic
