# Supplementary figures and images for: Nodding behavior couples to vigilance fluctuation in a high-calorie diet model of drowsiness
Source: Mol Brain. 2018 Jun 7;11:33. doi: 10.1186/s13041-018-0377-4 (PMC5992632; doi:10.1186/s13041-018-0377-4)

Figure S1

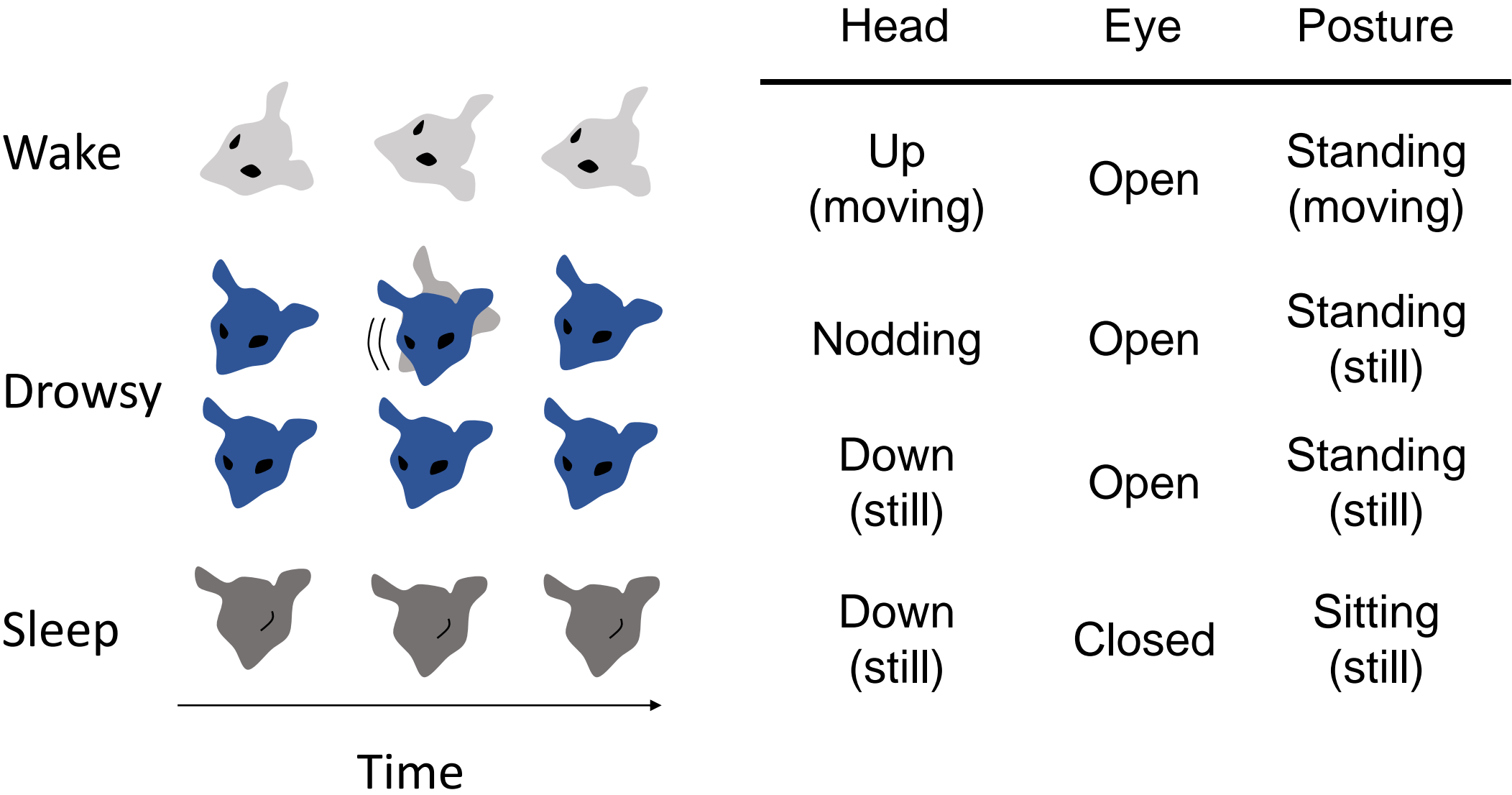

Supplement: Supplementary file 1 — Figure S1. Behavioral measurement according to vigilance level. Comparison of head/eye condition according to the behavior patterns. (PDF 92 kb) [file 13041_2018_377_MOESM1_ESM.pdf]

Figure S2

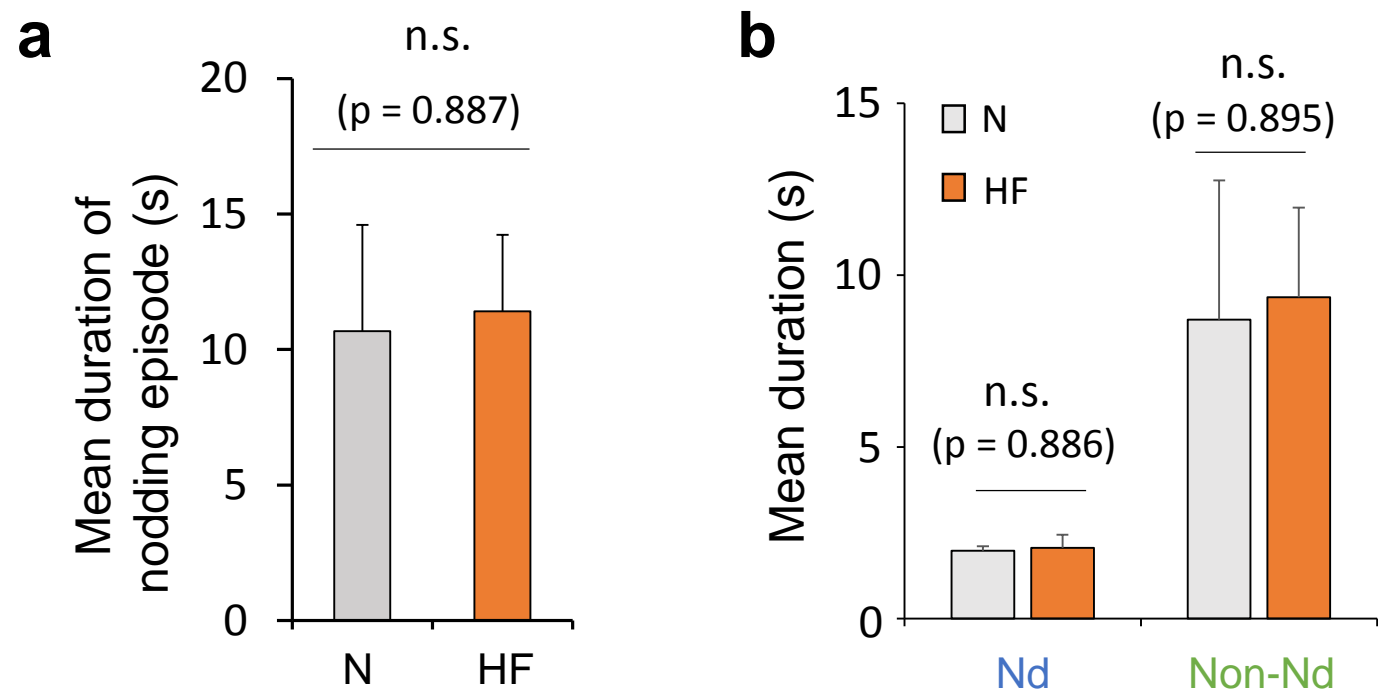

Supplement: Supplementary file 2 — Figure S2. Analysis of HF effects on drowsy states including nodding behavior. (a) The average length of nodding episode in the no-food and high-fat food groups (unpaired t-test, p = 0.887, no food n = 2 and high-fat n = 4, n.s. indicates ‘not significant’). All error bars represent s.e.m. (b) The mean duration of no food and high-fat food groups during Nd (unpaired t-test, p = 0.886) and Non-Nd states (unpaired t-test p = 0.895, no food n = 2 and high-fat n = 4). All error bars represent s.e.m. (PDF 88 kb) [file 13041_2018_377_MOESM2_ESM.pdf]
